# Dissolved Oxygen Concentration Prediction Model Based on WT-MIC-GRU—A Case Study in Dish-Shaped Lakes of Poyang Lake

**DOI:** 10.3390/e24040457

**Published:** 2022-03-25

**Authors:** Dianwei Chi, Qi Huang, Lizhen Liu

**Affiliations:** 1 School of Artificial Intelligence, Yantai Institute of Technology, Yantai 264003, China; chidianwei@yitsd.edu.cn; 2 Key Laboratory of Poyang Lake Wetland and Watershed Research, Ministry of Education, School of Geography and Environment, Jiangxi Normal University, Nanchang 330022, China; 3 Key Laboratory of Watershed Eco-Geological Processes, Ministry of Natural Resources, Nanjing 210016, China; 4 Institute of Microbiology, Jiangxi Academy of Sciences, Nanchang 330096, China; liulizhen@jxas.ac.cn

**Keywords:** dissolved oxygen prediction, dish-shaped lake, wavelet transform, maximal information coefficient, gated recurrent unit

## Abstract

Dissolved oxygen concentration has the characteristics of nonlinearity, time series and instability, which increase the difficulty of accurate prediction. In order to accurately predict the dissolved oxygen concentration in the dish-shaped lakes in Poyang Lake of Jiangxi Province, China, a dissolved oxygen concentration prediction model, based on wavelet transform (WT)-based denoising, maximal information coefficient (MIC)-based feature selection, and the gated recurrent unit (GRU), was proposed for this study. In experiments, the proposed model showed good prediction performance, achieving a root-mean-square error (RMSE) of 0.087 mg/L, a mean absolute percentage error (MAPE) of 0.723%, and a coefficient of determination (R2) as high as 0.998. It shows that the prediction model based on the combination of the wavelet transform and the GRU has a relatively high prediction accuracy and a better fitting effect. The model proposed in this study can provide a reference for protecting this type of lake-water body and the restoration of missing values in lake water quality monitoring data.

## 1. Introduction

Water quality prediction remains a fundamental task in water quality evaluation, management and protection. Advanced technologies, such as the Internet, Internet of things, and intelligent sensors, have been increasingly applied to water quality monitoring. They provide powerful tools for fast and real-time acquisition of water quality indicators and hence facilitate accurate prediction of changes in water quality, which is of great importance for establishing a water quality early-warning system [1]. The water environment of lakes is an unstable system subject to the impacts of climate changes, variations in the river basins, and socio-economic and human activities [2]. Establishing a prediction model based on water quality indicators can help us better understand the internal mechanisms of changes in the water environment, which is of great importance for water quality management and protection, as well as the prevention of water pollution. The water quality of lakes shows gradual, nonlinear, and uncertain changes [3], and macroscopically, seasonal and periodical variations, which are hard to simulate by conventional methods or classical mathematical models.

At the present time, as the online monitoring capacity and computing power for water quality data improve, data-driven models are seeing wider adoption in water quality prediction. The dissolved oxygen concentration is a crucial indicator of water quality and ecological well-being of lakes [4,5], and the accurate prediction of dissolved oxygen concentration plays an important role in monitoring and managing the water quality in lakes. As the studied area and the type of water bodies vary, the prediction model differs as well. Many existing works have adopted machine-learning methods, such as the support vector machine (SVM), multivariate adaptive regression splines (MARS), neural networks (long short-term memory network, generalized regression neural network, and backpropagation neural network), and polynomial chaos expansion to predict the dissolved oxygen concentration in surface water bodies, such as rivers, lakes and ponds [6,7,8,9,10,11,12,13,14,15,16,17,18]. For example, a multivariate adaptive regression spline (MARS) model using running water to predict dissolved oxygen concentration was proposed, and when compared with many machine-learning methods, it achieved better prediction results [19]. A feed forward neural network (FFNN) model and a radial basis function neural network (RBFNN) mode were proposed to predict the dissolved oxygen concentration of the Surma River, Bangladesh [20]. An Adaptive neuro-fuzzy inference system (ANFIS) was proposed to estimate accurately the biochemical oxygen demand (BOD) of the Surma River in Bangladesh [21], and was successfully applied to establish the river water quality prediction model. It was reported that the SVM performed better at dissolved oxygen prediction than the backpropagation (BP) neural networks, the generalized regression neural network (GRNN), MARS, and the M5 model tree [7,8,16]. A hybrid model that combined an autoregressive integrated moving average (ARIMA) with a support vector regression (SVR) was proposed in [14], which supplemented the nonlinear changes by the SVR; the model was trained on the samples of measured pH and dissolved oxygen concentration of Chaohu Lake in Anhui province, China, in 2004–2015, and achieved a high prediction accuracy. A hybrid MIC-SVR method was proposed in [10], which achieved an accurate prediction of dissolved oxygen in the Pearl River Basin, with a coefficient of determination (R2) of 0.9; they also found that using the MIC method could considerably reduce the error and improve the goodness of fit. Antanasijević et al. estimated the dissolved oxygen concentration of the Danube in northern Serbia by different neural networks, and found that the recurrent neural network (RNN) performed better than the GRNN and BP neural networks [13]. Since the online-monitored dissolved oxygen concentration data are time-series data, the RNN model is suitable for time-series processing, but they are prone to vanishing and exploding gradients when applied to a long time series [22].

Long short-term memory neural network (LSTM), a type of RNN [23,24], is specially designed to prevent the neural network output, for a given input, from either decaying or exploding as it cycles through the feedback loops. It can select memories, and the neurons in the network are controlled by three gates: input gate, output gate, and forget gate, so that the model can prevent the vanishing gradient problem and estimate the time-series variables more accurately than conventional RNNs. It can dig deep into the inherent laws of time series and learn long-term dependencies. However, the LSTM neural network has a complex structure and many parameters, so training and prediction are not efficient. The LSTM model can also be combined with data preprocessing methods, such as principal component analysis (PCA), K-similarity, and wavelet transform, to denoise the data and improve the prediction accuracy [25,26,27,28]. Liu et al. proposed a multi-factor water quality prediction model that denoised the input data by K-similarity and performed prediction by the LSTM model [26]; their model produced more accurate prediction results than the RNN and conventional LSTM models. GRU, a popular and streamlined variant of LSTM, has fewer parameters and simpler structures than the conventional LSTM models, and hence can converge faster and achieve better prediction than other LSTM variants.

Poyang Lake receives water from the basin and is directly connected to the Yangtze River. The water level changes with seasonal changes, and there are a large number of dish-shaped lakes. The special geomorphological and hydrological characteristics cause the dish-shaped lakes to play an increasingly important role in the basin ecosystem [29]. The dish-shaped lakes are connected to the main lake of Poyang Lake when the water level is high (summer and autumn), and form independent dish-shaped lakes when the water level falls (winter and spring). This unique environment means the dissolved oxygen in the water is affected by environmental factors, which are characterized by uncertainty and instability. Even though dissolved oxygen is an important and direct indicator of the health level of natural water ecosystems, there is minimal high-frequency and automatic water quality monitoring equipment deployed in the field due to cumbersome maintenance requirements and other factors, and the research is not in-depth, especially for complex and changeable water quality. There are relatively few studies on the change of dissolved oxygen concentration in the natural water body of dish-shaped lakes. In addition, long-term water quality monitoring data is easily affected by factors such as equipment and weather, and there is a certain amount of noise data, which affects the training speed and performance of the model. In order to accurately predict the dissolved oxygen concentration in the water body of the dish-shaped lakes in Poyang Lake, and to provide a scientific decision-making basis for the monitoring, management and maintenance of the water quality of Poyang Lake and its watershed, a dissolved oxygen concentration prediction model for lakes, by combining the wavelet transform (WT)-based denoising method, the maximal information coefficient (MIC)-based feature selection and the gated recurrent unit (GRU), was proposed for this study. Specifically, the WT method was employed to denoise the input data; then, the MIC method was used to calculate the correlation between each feature and the classification label, and features with high correlations were selected as the training features; finally, the GRU was used for model training. Furthermore, this proposed model is compared with three other models (including LSTM, GRU, GRU-WT), and the comparison results and the merits of the proposed model in this study are discussed. The proposed model obtained reliable sample data through data cleansing and denoising, and streamlined the prediction model through feature selection, which not only improved the training speed and accuracy, but also avoided overfitting while enhancing the model’s generalization capacity, providing a scientific decision-making basis for water quality monitoring, management and maintenance of Poyang Lake and its watershed.

The remainder of the paper is organized as follows. Section 2 introduces the concepts and theories related to the WT-MIC-GRU model, and then discusses the structure and flow of the model. Section 3 presents the source of the data sample set and various descriptive statistical metrics. Section 4 conducts experiments and discussions, including data denoising, feature selection, and model training phases. In order to illustrate the effectiveness of the model proposed in this paper, three baseline models are introduced for comparison, and the performance of each model is discussed through the experimental results.

## 2. Modelling

### 2.1. Wavelet Transform-Based Data Denoising

The model’s prediction performance depends on the authenticity and reliability of the data. The monitored water quality data, however, often have noise due to impacts from the devices and weather, so it is necessary to cleanse and denoise the sampled data prior to model training.

To reduce noise, we need to separate the signals from noise. Fourier analysis can differentiate signals in the frequency domain, but cannot analyze unstable signals, while the WT method can separate effective signals from noise by the differences between the two in the time domain and the frequency domain [30,31]. In this study, the WT method was employed to denoise the sampled data to maintain effective information while minimizing noise [32], so that temporal continuity and reliability of the sampled dissolved oxygen concentration data could be ensured. The WT method could decompose the original time-series signals into sub-signals to reveal as many time-series details of the original signals as possible. There are two common forms of wavelet transform, namely continuous wavelet transform (CWT), and discrete wavelet transform (DWT). Compared to the continuous wavelet transform, the discrete wavelet transform discretizes the scale and time, which can keep the reconstruction error low and save time and computing resources. Therefore, the DWT decomposition sequence is used in this paper.

The WT-based denoising steps are as follows.

1. The optimal wavelet functions for different feature variables are selected to decompose the signals. In this study, the Daubechies (db), Symlet (sym), Coiflet (coif) wavelet functions were selected.
2. The threshold is selected. Thresholds should be set to the high-frequency coefficients for quantification. A proper threshold should be set for each layer, and soft-thresholding is performed on high-frequency coefficients on each layer to smooth the signals.
3. The wavelets are restructured. The wavelets of the signals are restructured based on the high-frequency coefficient of each layer and the low-frequency coefficient of the last layer.
4. The denoising effect is evaluated. Two indicators, i.e., the signal-noise ratio (SNR) and the root-mean-square error (RMSE), are selected to evaluate the denoising effect. The wavelet function with a larger SNR and a smaller RMSE is considered to have better denoising performance.

### 2.2. Maximum Information Coefficient-Based Feature Selection

The monitored water quality data involve various feature parameters, which complicate the model, affecting the model’s training speed and prediction performance. Furthermore, the presence of features with weak or no relevance to the dissolved oxygen concentration will impair the model’s prediction accuracy, so dimensionality reduction should be performed on the features. There are two primary ways to reduce the dimensionality of features: feature transformation and feature selection [33].

The key to feature selection is constructing evaluation indicators for the sub-set of effective features based on features of high correlation to the dissolved oxygen concentration. There are three primary feature selection approaches: encapsulation, embedding and filtering [34].

The popular measures for the correlation between two features or between a feature and the labeled feature include the linear correlation coefficient, the chi-square and mutual information. The linear correlation coefficient indicates the closeness of the correlation between two variables and is a statistical measure widely used in many fields. The Pearson correlation coefficient is a correlation coefficient that gauges the linear correlation between two variables and is established based on the linear correlation between variable X and variable Y. The chi-square test is to test the correlation between the qualitative independent variable and the qualitative dependent variable; the mutual information is a measure of the correlation between two features with nonlinear relevance, but it applies only to the measurement of correlations between discrete variables. The features of the sampled water quality data of the lake in this study have no linear correlations, and all the features and dissolved oxygen concentration are quantitative and continuous. Thus, in this study, the MIC-based feature selection method was used to calculate the mutual information between features.

The MIC is a new measure proposed by Reshef et al. [35] to gauge the degree of nonlinear correlations between variables. The MIC-based method uses the maximal normalized mutual information to measure the degree of correlation between any feature and the target category, and applies the information theory and the idea of probability to continued data. In the MIC-based method, the joint probability density is used to measure the correlation between two random feature variables [36], which can measure the linear and nonlinear correlations between random variables, and hence can mine the internal correlations between variables. Besides, the MIC can label not only the discreteness of the eigenvalues, but the continuity of the values.

If two variables are correlated, the set of their corresponding data points will be distributed in 2D space. If the space is partitioned into m × n grids, there will definitely be a way to partition the scatter diagram of two variables. The MIC of variables x and y are defined as follows:

(1) $$\mathrm{MIC}\left(X;Y\right)=\max\left\{\frac{I\left(X;Y\right)}{\log\min\left\{n_{x},n_{y}\right\}}\right\}$$

where MIC(X;Y) represents the mutual information of X and Y, and n_x_ and n_y_ represent the number of segments of the variables X and Y during the grid partitioning process, respectively.

### 2.3. Construction of the WT-MIC-GRU Prediction Model

#### 2.3.1. Gated Recurrent Unit

The GRU shares the same input structure with RNN: when the current input $x_{t}$ and the cell state $h_{t-1}$ (calculated at the preceding time point) are input to the GRU, the two output states are the output of the current hidden node $y_{t}$ and the cell state $h_{t}$.

**Figure 1:**
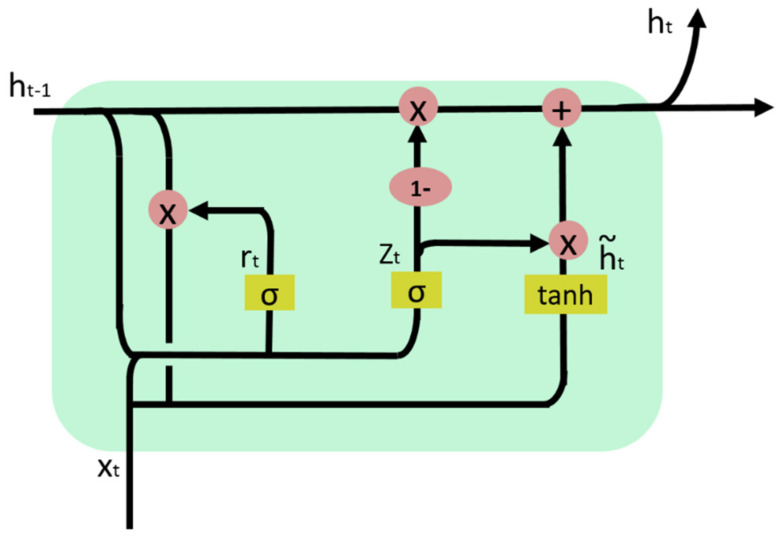
Internal structure of GRU.

Figure 1 shows the internal structure of the GRU.

There are two gates in a GRU: the reset gate and the update gate.

(1) Update gate

The update gate determines how much information will be transmitted to the next time step. The model can copy all previous information, which reduces the risk of vanishing gradient. At the time step t, Equation (2) is used to calculate the gated signal at the update gate:

(2) $$z_{t}=\sigma \left(W_{z}*\left[x_{t},h_{t-1}\right]\right)$$

where $x_{t}$ is the input vector at the time step t, which is multiplied by the weight matrix $W_{z}$ to perform linear transformation; $h_{t-1}$ stores the information obtained at the preceding time step (t − 1), which will also undergo linear transformation. Information from these two parts is summed and input to the sigmoid activation function, and the output is the gated signal that is between 0 and 1. The closer the gated signal approaches 1, the more the past information is memorized.

(2) Reset gate

The reset gate, which determines how much information to forget, is used to screen the current information. It involves three steps. The first step is to calculate the value of the reset signal $r_{t}$, and $W_{r}$ is the reset weight matrix:

(3) $$r_{t}=\sigma \left(W_{r}*\left[x_{t},h_{t-1}\right]\right)$$

The second step is to reset the state of the preceding cell to filter the information transmitted from the preceding step. The information obtained through resetting is ${h^{\prime }}_{t-1}$, the calculation equation for which is as follows:

(4) $${h^{\prime }}_{t-1}=h_{t-1}⨂r_{t}$$

Then, the current cell state $\tilde{h_{t}}$ is calculated, which means selectively memorizing the filtered information and the input of the current time step. In the equations, ⨂ means the multiplication of elements in the matrix, tanh is the activation function, W is the weight. The calculation equation for $\tilde{h_{t}}$ is as below:

(5) $$\tilde{h_{t}}=\tan h(W*\left[x_{t},{h^{\prime }}_{t-1}\right]$$

Last, the network calculates the current cell state $h_{t}$, which retains the information of the current unit and transmits it to the next unit. The equation is as follows.

(6) $$h_{t}=\left(1-z_{t}\right)⨂h_{t-1}+z_{t}⨂\tilde{h_{t}}$$

The ultimate output of the current cell is:

(7) $$y_{t}=\sigma (W_{0}*h_{t})$$

where $W_{0}$ is the weight matrix.

#### 2.3.2. WT-MIC-GRU Prediction Model

**Figure 2:**
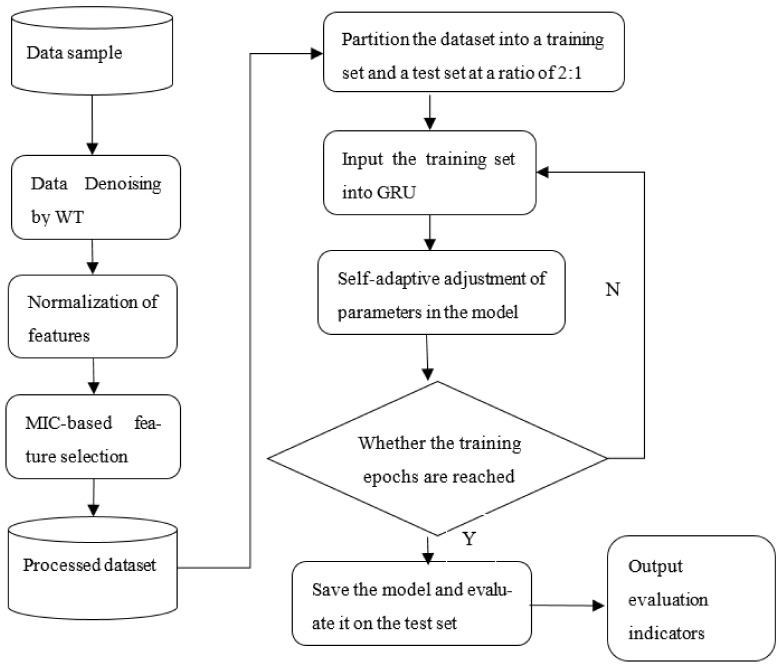
Flowchart of the WT-MIC-GRU prediction model.

A dissolved oxygen concentration prediction model for lake water based on WT-MIC-GRU is proposed in this study, and the specific prediction workflow is shown in Figure 2.

The WT-MIC-GRU prediction model was employed to predict the dissolved oxygen concentration of Poyang Lake. Specifically, the sample dataset was denoised by the WT method, and the eigenvalues of all features were normalized; then, the MIC was employed for feature selection, and features with a MIC ≥ 0.3 [10] were selected for dissolved oxygen concentration prediction; finally, the GRU model was trained and tested. The algorithm was configured as follows: the time step of GRU was set at 3, the number of hidden units was 32, the batch size was 100, the learning rate was 0.001, and the number of iterations was set at 50. Among all the sampled data, 67% was used as the training set, and the remaining 33% used as the test set to perform prediction.

## 3. Acquisition of Sample Data

The sample data used in this study are real-time monitored data from the dish-shaped lakes of Poyang Lake, and there are 11 monitoring indicators: atmospheric temperature, wind direction, wind speed, atmospheric pressure, relative humidity, water temperature, pH scale, conductivity, measured water depth, redox potential, and dissolved oxygen concentration. The monitoring time is from April to November 2017 (eight months); the data were sampled every two minutes, and a total of 7803 pieces of data were obtained. Descriptive statistical indicators of the sampled data are shown in Table 1.

In order to improve the quality of the monitored data, it is necessary to perform data cleaning on sample data before model training. The k-means clustering method is used to identify abnormal data. For problems, such as missing data, according to the characteristics of small samples and nonlinearity in the data, support vector regression is used to recover the missing data [37].

## 4. Results and Analysis

### 4.1. Data Pretreatment

Data pretreatment involves two steps: normalization of eigenvalues of the samples; and data denoising.

(1) WT-based data denoising

The model’s prediction performance relies on the authenticity and reliability of the sample data. The monitored water quality data of the lake, due to system errors, random errors and human errors, may be polluted by noise. Furthermore, the monitoring devices deployed underwater for long periods are likely to be affected by pollutants and are susceptible to the impacts of weather changes, which may produce data that deviate from reality. Therefore, it is necessary to denoise the sampled data. In this study, the WT-based denoising algorithm was employed to retain effective information and perform wavelet decomposition on the sample data; the decomposed wavelet coefficient was processed by the gate threshold, and the wavelet reconstruction was employed on the signals to reduce the noise. The principle of determining the number of decomposition levels is that at least one correct wavelet transform coefficient should be obtained when the decomposition reaches the maximum level. That is, at this time, the length of the stretched wavelet mother function should not be greater than the length of the signal to be analyzed to calculate the maximum number of layers. This ensures that the results are reasonable. This paper uses the dwt_max_level function of the PyWavelets analysis library to calculate the highest decomposition order that the signal can achieve. In this study, different wavelet functions were used to process the 11 feature variables to compare the denoising effect, the specific results are shown in Table 2.

Three wavelet functions, coif5, sym10, and db8, were used to denoise the feature variables. The two indicators, SNR and RMSE, were used to evaluate the denoising effect and select the optimal function for each feature variable. In the test, the global soft threshold was used as the threshold, set at 0.004. As per the denoising effect, the function with the minimum SRN and RMSE was identified as the optimal function, and hence the combinations of features and wavelet functions were as follows:

The sym10 function was used for the features of atmospheric temperature, atmospheric pressure, water temperature, pH scale, conductivity, redox potential, and dissolved oxygen concentration; the coif5 function was used for the features of wind direction, wind speed, relative humidity and measured water depth; and the db8 function was used for other features.

**Figure 3:**
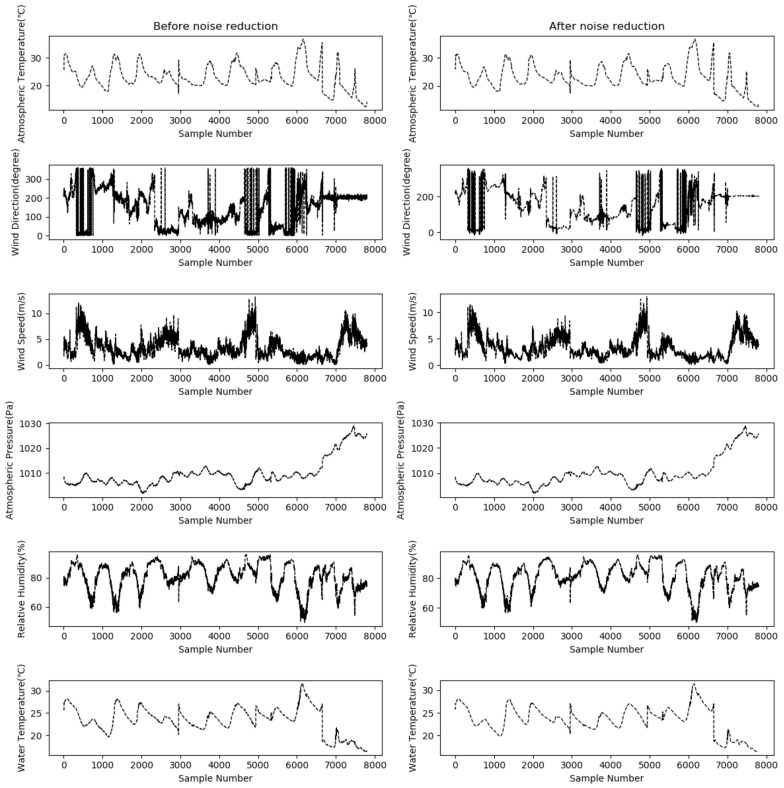
Denoising effect of data about the six features using corresponding wavelet functions.

After denoising, a dataset consisting of 7803 × 11 pieces of data was obtained, and the data of specific features were denoised by the corresponding optimal wavelet function, as mentioned above. Figure 3 shows the denoising results of data of the first six features (atmospheric temperature, wind direction, wind speed, atmospheric pressure, relative humidity, and water temperature). The figures on the left present the distribution of samples before denoising, and the figures on the right show the distribution of samples after denoising.

As can be seen from Figure 3, for each sample feature, after wavelet transform and noise reduction, there is a certain degree of smoothing. Among them, the numerical fluctuation of the wind direction feature is relatively large. After the noise reduction process, the signal-to-noise ratio is improved, the noise level is reduced to a certain extent, and the curve smoothing effect is obvious, thereby improving the accuracy and stability of the dissolved oxygen prediction model to a certain extent.

(2) Eigenvalue normalization.

Because many water quality indicators were selected for this study, the sample data consist of 10 variables that have different dimensionalities and differ greatly. To remove the impact of differences in the unit and scale of the features, the variables were normalized; that is, each feature was adjusted to a specific range. The max–min normalization was performed to transform all eigenvalues into values within the range of [0, 1] to reduce the fluctuation and complexity of data. The max–min normalization equation is presented below:

(8) $$x_{t}^{\prime }=\left(x_{t}-x_{\min}\right)/\left(x_{\max}-x_{\min}\right)$$

where x_max_ and x_min_ represent the maximum and minimum of the sample data for the same feature, x_t_ is the original value of the sample data, and $x_{t}^{\prime }$ is the normalized value.

### 4.2. Feature Selection

The features of the sample data obtained in the present work do not necessarily present a linear correlation with the feature of dissolved oxygen concentration, and the values of all features are quantitative and continued data. The MIC-based feature selection method was used to calculate the correlation between dissolved oxygen and other features, and the features with high correlations were used as the input features to the LSTM prediction model.

The variable of the dissolved oxygen concentration feature was set as Y, and that for other features as X. The steps of the MIC-based method are as follows.

1. The i and j were given, and scatter diagram composed by X and Y were partitioned into i columns and j lines, and the maximal mutual information was obtained;
2. The maximal mutual information was normalized;
3. The maximal mutual information under different scales was considered as the MIC value.

**Figure 4:**
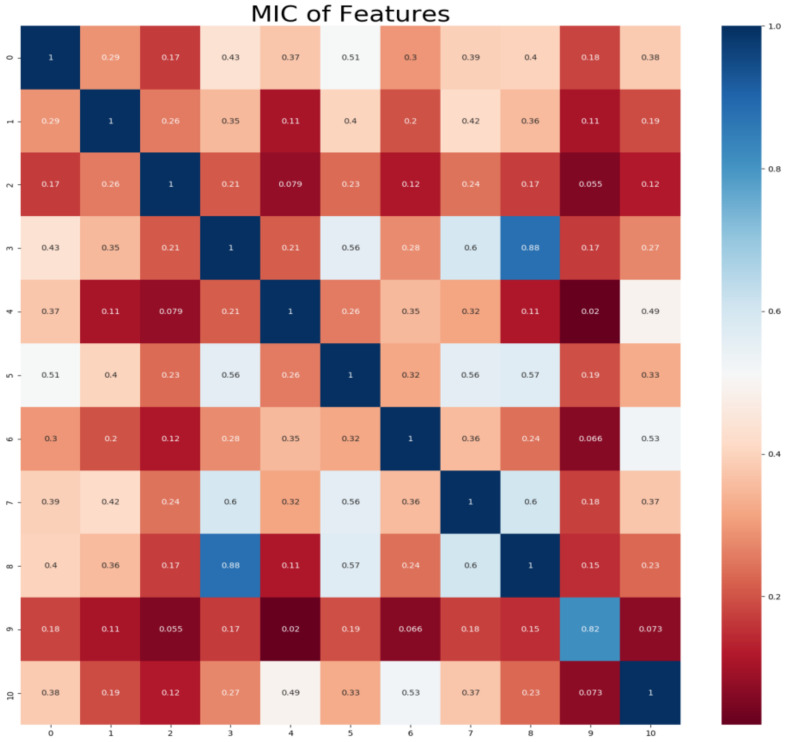
Correlation coefficient between sample features.

In this study, the monitored water quality dataset of the dish-shaped lake was used. The minepy library of Python was employed to calculate the MIC between different features, and the searborn library was utilized to visualize the MIC matrix between features, as shown in Figure 4.

Figure 4 shows the correlation coefficients between features in this study. Numbers 0–10 represent the 11 features of atmospheric temperature, wind direction, wind speed, atmospheric pressure, relative humidity, water temperature, pH scale, conductivity, measured water depth, redox potential and dissolved oxygen concentration, respectively. Table 3 lists the correlation coefficient between dissolved oxygen and other features obtained by the MIC algorithm.

As Table 3 shows, the pH scale and relative humidity have the largest correlation with dissolved oxygen. To reduce the computation overhead of the LSTM model and improve its generalization capacity, the threshold of MIC was set at 0.3 [10], and variables that have little correlation to the target variable (dissolved oxygen) were removed (MIC < 0.3). Consequently, the features used for model training were reduced to four features: atmospheric temperature, relative humidity, pH scale, and conductivity.

### 4.3. GRU Model Training and Evaluation

The sample data, after denoising and feature extraction, were transmitted to the GRU model for training. The GRU model was optimized by the Adam algorithm [38]. The Adam algorithm combines the advantages of Adagrad for dealing with sparse gradients and RMSProp for dealing with non-stationary targets, and calculates different adaptive learning rates for different parameters. A learning rate was set to update the weight, and the test set was used to test the model’s performance.

To reflect the mean error between the predicted value and the measured value, the root-mean-square-error (RMSE) was used to evaluate the model’s performance. RMSE is the arithmetic square root of the mean error, while the mean square error (MSE) is the expected value of the error between the predicted value and the measured value. Equation (9) shows the calculation equation of MSE, where y_i_ is the measured value, and *p*_i_ is the predicted value.

(9) $$\mathrm{MSE}=\frac{1}{N}\overset{N}{\underset{i=1}{\sum }}{\left(y_{i}-p_{i}\right)}^{2}$$

RMSE is the square root of Equation (9), which can well describe the deviation of the predicted results from the reality, the unit of which is the same as that of the dataset. A smaller RMSE indicates a more stable model.

The model’s accuracy and fitting effect were assessed by the mean absolute percentage error (MAPE) and coefficient of determination (R2). MAPE represents the ratio of the absolute value of error of all samples to the measured value. The closer the MAPE approaches 0, the more accurate the model is. The calculation equation of MAPE is:

(10) $$\mathrm{MAPE}=\frac{1}{N}\overset{N}{\underset{i=1}{\sum }}\frac{\left|y_{i}-{\hat{y}}_{i}\right|}{\left|y_{i}\right|}$$

where y_i_ is the measured value, and ${\hat{y}}_{i}$ is the predicted value.

The coefficient of determination, i.e., R2, represents the proportion of variance in the dependent variable that can be explained by the independent variable. It indicates the model’s fitting effect, and the range is set in [0, 1]. The larger the R2 is, the better the fitting effect of the model. The calculation equation of R2 is shown in Equation (11):

(11) $$R^{2}=1-\frac{\sum_{i=1}^{N}{\left(y_{i}-{\hat{y}}_{i}\right)}^{2}}{\sum_{i=1}^{N}{\left(y_{i}-{\bar{y}}_{i}\right)}^{2}}$$

where $y_{i}$ is the measured value, and ${\bar{y}}_{i}$ is the mean value of the samples.

In this paper, Willmott’s Index of Agreement (WIA) is used to evaluate the generalization performance of the model, and the calculation is shown in Equation (12):

(12) $$\mathrm{WIA}=1-\frac{\sum_{i=1}^{N}{\left(y_{i}-{\hat{y}}_{i}\right)}^{2}}{\sum_{i=1}^{N}{\left(|y_{i}-\bar{y}\left|+\right|{\hat{y}}_{i}-\bar{y}|\right)}^{2}}$$

### 4.4. Comparative Experiments

To verify the effectiveness of the proposed model, a proposed model that combines WT-based denoising, MIC-based feature selection and GRU was compared with three baseline models (LSTM, GRU, GRU-WT) by experiments. Table 4 shows the results.

As Table 4 shows, GRU achieves higher accuracy than LSTM, with the R2 increased from 0.954 to 0.996, and the RMSE reduced by 0.343. That is, the GRU model has improved the dissolved oxygen prediction accuracy by 72.8% on average, and reduced the MAPE from 1.495% to 0.712%. This means the GRU model has a higher prediction accuracy and better fitting effect than the LSTM model.

Compared with the conventional GRU model, the WT-MIC-GRU model further improves the R2 to as high as 0.998 and reduces the RMSE by 0.041. That is, our proposed model achieves an accuracy 32.03% higher than that of the conventional GRU model, which means that our model has considerably improved the dissolved oxygen prediction accuracy. Compared with the conventional GRU model, the “WT-GRU” model that introduced WT to denoise the data, reduced the MAPE from 0.712% to 0.666%, indicating that using the dataset processed by WT for training would achieve a model with better fitting effect. Compared with the “WT-GRU” model, however, our model that introduced the MIC method further improved the MAPE from 0.666 to 0.723, which suggests that the MIC-based feature selection has a positive impact on the fitting effect. Judging by all the evaluation indicators, our model proved to be the best model among all the models compared.

From the WIA results of each comparative model, the GRU model has a certain improvement in the WIA of the LSTM model, indicating that it has better generalization ability; that is, a stronger prediction ability. The WIA of the model proposed in this paper reaches 1.0, which is the best among all models.

There are many combination methods based on the LSTM model, and some studies have achieved good prediction results. For example, Chi Dianwei et al. proposed a model based on the combination of principal component analysis (PCA), maximal information coefficient and long short-term memory neural network (LSTM) to predict the dissolved oxygen content of the dish-shaped lake [39], and achieved good predictions. The coefficient of the determination reached 0.99. Sun Longqing et al. proposed a prediction model of dissolved oxygen content in pond water, based on IBAS and LSTM networks [40]; the root-mean-square error of the model was 0.8026, and it had good generalization performance; In Chen Yingyi et al., the dissolved oxygen content prediction model of CNN-LSTM [41] achieved good results in predicting the dissolved oxygen content in aquaculture after 2 h. The model root-mean-square error was 0.229, and the coefficient of determination was 0.954. The above three models are all combined models based on LSTM, but their accuracy and coefficient of determination cannot match the performance of the model proposed in this paper. In addition, the GRU model has a simpler network structure than the LSTM model, which can make the parameters converge faster, reduce the possibility of overfitting to a certain extent, and have better prediction effects on certain tasks, which can meet the forecasting needs of larger data samples size with longer time series.

**Figure 5:**
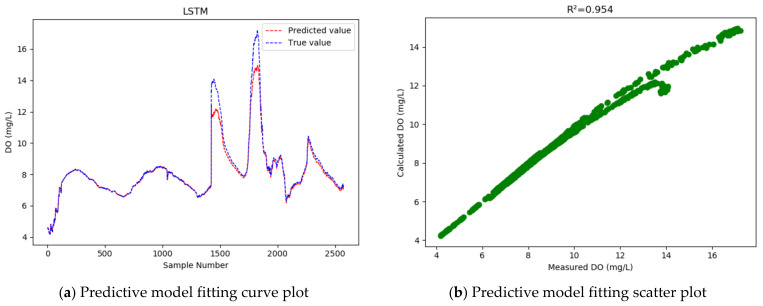
Dissolved oxygen prediction by the LSTM model.

**Figure 6:**
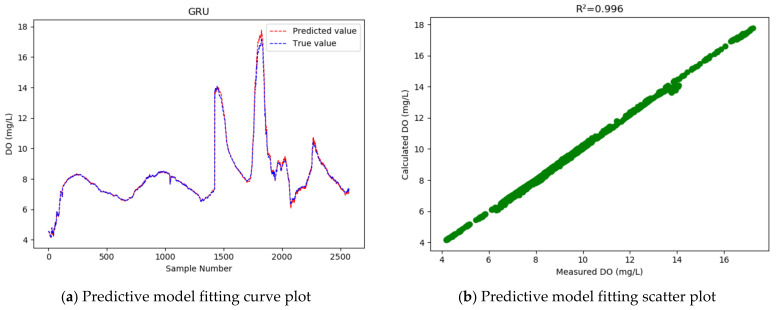
Dissolved oxygen prediction by the GRU model.

**Figure 7:**
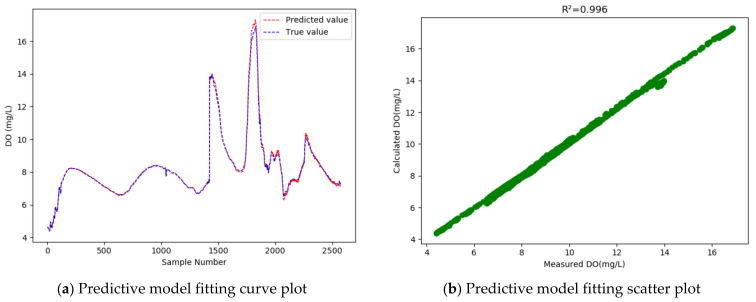
Dissolved oxygen prediction by the WT-GRU model.

**Figure 8:**
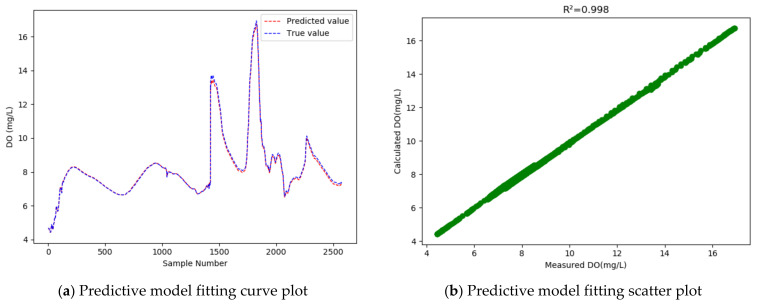
Dissolved oxygen prediction by the WT-MIC-GRU Model.

In our experiment, 33% of the samples were used as the test set to test the models, and then a curve line was drawn, according to the predicted value and the actual value of the test sample data, where the abscissa represents the serial number of the test sample point, and the ordinate represents the dissolved oxygen concentration value. Figure 5, Figure 6, Figure 7 and Figure 8 show the fitted curve of the predicted value and the actual value of each model, and the relationship between these two values.

As the fitting curves of the predicted dissolved oxygen concentration and the true dissolved oxygen concentration show (Figure 5, Figure 6, Figure 7 and Figure 8), the GRU model has a better fitting effect than LSTM, and the WT-MIC-GRU model further improves the fitting effect than the conventional GRU model without data denoising and feature selection. The presence of noise in the sample data will impair the model’s prediction accuracy. Our proposed WT-MIC-GRU model, however, effectively avoids the impact of noise and achieves a high prediction accuracy; meanwhile, with the features highly correlated to the dissolved oxygen concentration, identified by the MIC-based method as the inputs to the LSTM model, our model has reduced the computation complexity and achieved a better fitting effect. In conclusion, the model prediction proposed in this paper is relatively optimal in terms of stability, accuracy and fitting effect, and is an effective method for predicting the dissolved oxygen concentration of dish lake water. In order to improve the prediction model in the future, the equipment that collects the data could be fitted with special cleaning equipment to ensure that the data can reduce noise and redundancy from the root cause.

The data set used in this paper is based on the real-time online monitoring data of the dish-shaped lakes. Poyang Lake is the largest freshwater lake in China and is directly connected to the Yangtze River. High water level fluctuations lead to numerous dish-shaped lakes, which are connected to Poyang Lake at its high water level in summer and autumn, and form independent dish-shaped lakes in winter and spring. This unique phenomenon can predict the dissolved oxygen in this district to be more complex. As shown in Table 1, the dissolved oxygen in a dish-shaped lake is extremely unstable. The range can reach 13.06 mg/L, which is higher than the range reported for deep lakes and shallow lakes [42,43]. The WT-MIC-GRU model is a prediction model of dissolved oxygen concentration proposed for the unique dish-shaped lake, and experiments have proved its excellent prediction performance. Among them, the noise reduction and MIC-feature extraction, based on wavelet transform, can significantly improve the stability and accuracy of the model, and because the parameters of the GRU model are simplified compared to the LSTM model, the prediction efficiency is higher, and is suitable for larger-scale data prediction. Therefore, the WT-MIC-GRU model is useful for carrying out and improving the water quality monitoring and protection of such lakes.

At the same time, judging from the actual observed value distribution in Figure 5, Figure 6, Figure 7 and Figure 8, there are two places between the sample point serial numbers 1500–2000 where the dissolved oxygen concentration fluctuates greatly, and the fitting effect is not good. The dish-shaped lakes are connected to the main lake of Poyang Lake when the water level is high in summer and autumn, and form independent dish-shaped lakes in winter and spring. This unique environment means the dissolved oxygen in the water is affected by environmental factors, which are characterized by uncertainty and instability. Both long-term trends and seasonal effects, and the two places mentioned above, happen to coincide with the change of seasons, and this sharp fluctuation will have a certain impact on the prediction results of the time series-based recurrent neural network model. In the future, we will consider increasing the training sample data set to further improve the generalization ability of the model; and we will consider adding seasonal factors to the model construction, to provide stronger adaptability near the seasonally alternating sample points.

## 5. Conclusions

In view of the many and complex factors affecting the dissolved oxygen in dish-shaped lake water, combined with its time series and nonlinear characteristics, a WT-MIC-GRU model for predicting the dissolved oxygen concentration in dish-shaped lake water is proposed. Among them, WT noise reduction and MIC-feature extraction processing can improve the reliability of data and reduce the complexity of the model, thereby significantly improving the stability, accuracy and generalization ability of the model. The proposed model was compared with LSTM, GRU, and the GRU-WT models in experiments. The following major conclusions were reached:

1. Compared with the LSTM model, the GRU model achieved higher accuracy in the prediction of the dissolved oxygen concentration in the dish-shaped Poyang Lake, with the coefficient of determination increased from 0.954 to 0.996; meanwhile, the RMSE was reduced by 0.343, and the MAPE dropped from 1.495% to 0.712%, indicating that the GRU model achieves a better fitting effect than LSTM.
2. The GRU model, after introducing WT method for data denoising and the MIC method for feature selection, increased the R2 of the conventional GRU model from 0.996 to 0.998, and reduced the RMSE by 0.041, indicating improved prediction accuracy. It also indicates that data denoising and feature selection could considerably improve the model’s performance.
3. The GRU model that incorporated the WT for data denoising, but not feature selection, achieved an MAPE of 0.666%, and when the feature selection method was introduced, the MAPE rose to 0.723%, which means that feature selection had a positive impact on the fitting effect. Judging by all the evaluation indicators, our proposed model achieved the best performance among all models that were compared.

Our study still has some limitations, which will be improved in future research. On the one hand, the data time span used in the training of the model proposed in this paper is 8 months, and it does not contain extreme period data, which affects the generalization ability of the model. In future research, more years of data will be considered to enhance the applicability and generalization ability of the model. On the other hand, although the GRU model works well on long time-series problems, it does not distinguish the information of each time step of the long time series, and may ignore some time-series nodes that have a significant impact on the final prediction results. Therefore, in future research, the attention mechanism based on time series will be considered to highlight the influence of different nodes on dissolved oxygen, thereby improving the performance of the model.

## Figures and Tables

**Table 1:** Descriptive statistical indicators of sampled data.

	Atmospheric Temperature (°C)	Wind Direction (Degree)	Wind Speed (m/s)	Atmospheric Pressure (KPa)	Relative Humidity (%)	Water Temperature (°C)	pH (/)	Conductivity (µS/cm)	Measured Water Depth (m)	Redox Potential (mv)	Dissolved Oxygen Concentration (mg/L)
Mean	23.11	144.42	3.37	1009.9	81	23.46	6.87	107.14	0.36	0.27	7.48
Maximum	36.89	359	13.2	1029	96	31.56	9.18	151.9	0.63	−0.1	17.23
Minimum	12.28	0	0.03	1001.7	49	16.4	5.79	93.2	0.25	−0.4	4.17
Standard deviation	4.55	92.7	2.10	5.75	9.37	3.01	0.38	15.72	0.07	0.04	1.53
Coefficient of variation	19.69%	64.19%	62.31%	0.57%	11.57%	12.83%	5.53%	14.67%	19.44%	14.81%	20.45%

**Table 2:** Comparison of denoising effect of different functions on 11 feature variables.

Feature Variables	Evaluation Indicators	Coif5	Sym10	Db8
Atmospheric temperature	SNR/db	25.976	27.162	23.85
	RMSE	0.225	0.196	0.282
Wind direction	SNR/db	19.354	19.295	18.383
	RMSE	9.623	9.693	10.655
Wind speed	SNR/db	21.667	20.58	20.79
	RMSE	0.169	0.191	0.184
Atmospheric pressure	SNR/db	36.214	36.936	35.494
	RMSE	0.089	0.082	0.096
Relative humidity	SNR/db	24.052	23.956	22.325
	RMSE	0.577	0.583	0.683
Water temperature	SNR/db	29.468	30.894	27.133
	RMSE	0.101	0.085	0.13
pH scale	SNR/db	19.413	22.115	19.162
	RMSE	0.039	0.029	0.039
Conductivity	SNR/db	28.376	28.56	26.704
	RMSE	0.597	0.585	0.722
Measured water depth	SNR/db	33.009	32.887	31.849
	RMSE	0.002	0.002	0.002
Redox potential	SNR/db	18.102	18.271	17.947
	RMSE	0.005	0.005	0.005
Dissolved oxygen	SNR/db	20.926	22.161	19.233
	RMSE	0.134	0.116	0.159

**Table 3:** Correlation coefficients between dissolved oxygen and other eigenvalues by the MIC algorithm.

Features	Correlation with Dissolved Oxygen
Atmospheric temperature	0.38
Wind direction	0.19
Wind speed	0.12
Atmospheric pressure	0.27
Relative humidity	0.49
Water temperature	0.33
pH scale	0.53
Conductivity	0.37
Measured water depth	0.23
Redox potential	0.073

**Table 4:** A comparison of our proposed model with another standalone model.

Model	RMSE	MAPE%	R2	WIA
LSTM	0.471	1.495%	0.954	0.986
GRU	0.128	0.712%	0.996	0.999
GRU-WT	0.126	0.666%	0.996	0.999
WT-MIC-GRU	0.087	0.723%	0.998	1.000

## Data Availability

The data supporting the study can be found in the article.
